# (1*H*-Pyrazole-κ*N*)bis­(tri-*tert*-but­oxy­silane­thiol­ato-κ^2^ *O*,*S*)cadmium

**DOI:** 10.1107/S1600536812047186

**Published:** 2012-11-24

**Authors:** Katarzyna Baranowska, Mateusz Bulman, Anna Dołęga

**Affiliations:** a Department of Inorganic Chemistry, Faculty of Chemistry, Gdansk University of Technology, 11/12 G. Narutowicz St., 80233 - PL Gdańsk, Poland

## Abstract

The Cd^II^ atom in the title complex, [Cd(C_12_H_27_O_3_SSi)_2_(C_3_H_4_N_2_)], is penta-coordinated by two O and two S atoms from the *O*,*S*-chelating silane­thiol­ate residue and one pyrazole N atom in a distorted geometry that is slightly closer to trigonal–bipyramidal than to square-based pyramidal. The pyrazole ligand is stabilized within the complex by an intra­molecular N—H⋯O hydrogen bond. One of the *tert*-butyl groups is disordered over two orientations with occupancy ratio of 0.534 (6):0.466 (6).

## Related literature

For similar compounds, see: Dołęga *et al.* (2005 ▶, 2006 ▶, 2008 ▶, 2009 ▶); Dołęga (2010 ▶). For the synthetic procedure, see: Pladzyk *et al.* (2011 ▶). For a description of the geometry of complexes with five-coordinate metal atoms, see: Addison *et al.* (1984 ▶).
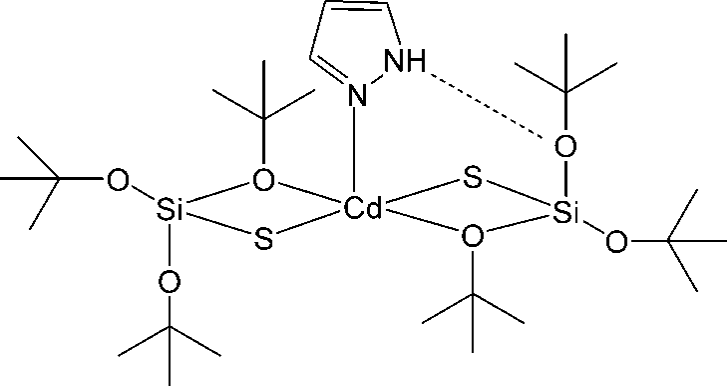


## Experimental

### 

#### Crystal data

[Cd(C_12_H_27_O_3_SSi)_2_(C_3_H_4_N_2_)]
*M* *_r_* = 739.45
Triclinic, 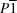

*a* = 9.4445 (4) Å
*b* = 12.7322 (4) Å
*c* = 16.9153 (5) Å
α = 109.098 (3)°
β = 92.905 (3)°
γ = 96.050 (3)°
*V* = 1903.53 (12) Å^3^
*Z* = 2
Mo *K*α radiation
μ = 0.78 mm^−1^
*T* = 120 K
0.2 × 0.18 × 0.08 mm

#### Data collection

Agilent Xcalibur Sapphire2 diffractometer
11378 measured reflections
7083 independent reflections
5935 reflections with *I* > 2σ(*I*)
*R*_int_ = 0.025

#### Refinement

*R*[*F* ^2^ > 2σ(*F* ^2^)] = 0.039
*wR*(*F* ^2^) = 0.103
*S* = 1.06
7083 reflections
414 parameters
H atoms treated by a mixture of independent and constrained refinement
Δρ_max_ = 1.11 e Å^−3^
Δρ_min_ = −0.65 e Å^−3^

### 

Data collection: *CrysAlis PRO* (Agilent, 2010 ▶); cell refinement: *CrysAlis PRO*; data reduction: *CrysAlis PRO*; program(s) used to solve structure: *SUPERFLIP* (Palatinus & Chapuis, 2007 ▶); program(s) used to refine structure: *SHELXL97* (Sheldrick, 2008 ▶); molecular graphics: *ORTEP-3 for Windows* (Farrugia, 2012 ▶); software used to prepare material for publication: *WinGX* (Farrugia, 2012 ▶).

## Supplementary Material

Crystal structure: contains datablock(s) global, I. DOI: 10.1107/S1600536812047186/vn2060sup1.cif

Structure factors: contains datablock(s) I. DOI: 10.1107/S1600536812047186/vn2060Isup2.hkl

Additional supplementary materials:  crystallographic information; 3D view; checkCIF report

## Figures and Tables

**Table 1:** Selected bond lengths (Å)

Cd1—N1	2.298 (3)
Cd1—O1	2.536 (2)
Cd1—O4	2.649 (2)
Cd1—S1	2.4438 (8)
Cd1—S2	2.4503 (8)
Si1—S1	2.0917 (11)
Si2—S2	2.0781 (12)

**Table 2:** Hydrogen-bond geometry (Å, °)

*D*—H⋯*A*	*D*—H	H⋯*A*	*D*⋯*A*	*D*—H⋯*A*
N2—H2⋯O5	0.83 (4)	2.14 (4)	2.959 (3)	167 (4)

**Fig. 1.**
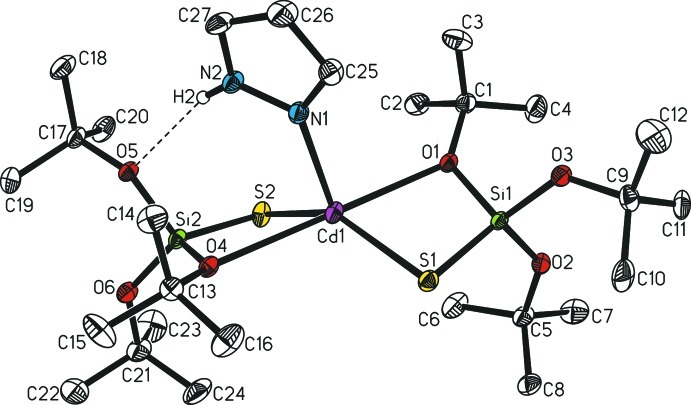
A view of the molecule of (I), showing the atom-labelling scheme. Displacement ellipsoids are drawn at the 30% probability level and H atoms have been omitted. Hydrogen bond indicated with dashed lines. Only the major conformation for the disordered tert-butyl group (C10—C12) is shown.

**Fig. 2.**
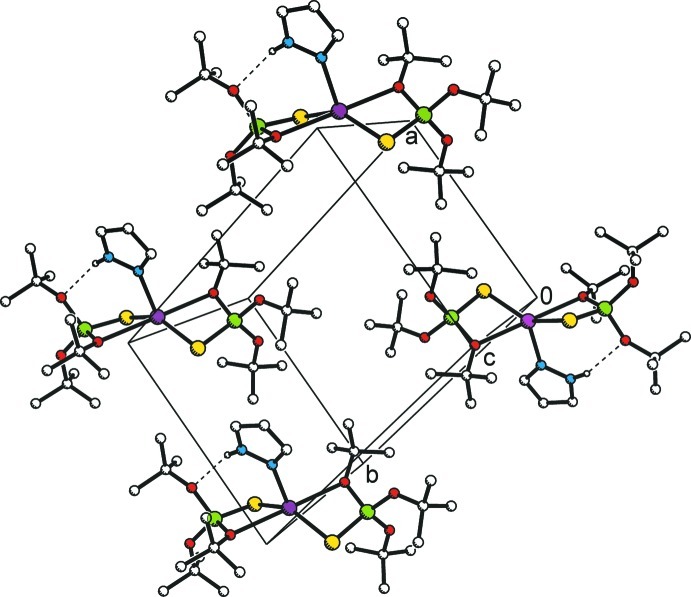
Crystal packing of the title compound. Hydrogen bonds are indicated with dashed lines.

## supplementary crystallographic information

### Comment

Our current research subject concerns the structural and spectroscopic characteristics of simple inorganic complexes - models of the catalytic site of the enzyme alcohol dehydrogenase. We use tri-*tert*- butoxysilanethiol as a source of thiolate function and aromatic N-ligands as histidine analogs (Dołęga, 2010 and references therein)

The title complex is the first heteroleptic cadmium(II) tri-*tert*-βutoxysilanethiolate with the pyrazole co-ligand. The NH group of pyrazole ligand forms an intramolecular hydrogen bond with oxygen and there are no other strong intra- or intermolecular interactions in the crystal other than van der Waals. Therefore with regard to the crystal packing the title compound resembles rather analogous pyridine-containing zinc and cadmium complexes than imidazole-containing analogs (Dołęga *et al.*, 2005; Dołęga *et al.*, 2006; and Dołęga *et al.* 2009). The coordination geometry is in between square-base pyramidal and trigonal-bipiramydal, but closer to the latter based on the calculation of the τ-parameter (0.597; Addison *et al.*, 1984). The cadmium-ligand bond distances are comparable to those found in similar complexes (Dołęga *et al.*, 2008; Dołęga *et al.*, 2009). However, the single intramolecular NH···O hydrogen bond introduces further asymmetry into the molecule, including the bond lengths (*i.e.* Cd—O1 and Cd—O4, see Table 1). The title complex features a characteristic, large (> 140°) S—Cd—S angle, and an O—Cd—O angle close to 180°.

The molecular structure of (I) is shown in Fig. 1 and a crystal packing diagram is presented in Fig. 2.

### Experimental

Compound (I) was crystallized at low temperature (255 K) from toluene-methanol solution of cadmium bis(tri-*tert*-butoxysilanethiolate) (synthesis described in Pladzyk *et al.*, 2011) and pyrazole.

### Refinement

All C–H hydrogen atoms were refined as riding on carbon atoms with methyl C–H = 0.98 Å, aromatic C–H = 0.95 Å and *U*_iso_(H)=1.2 *U*_eq_(C) for aromatic and 1.5 *U*_eq_(C) for methyl groups. Atoms C10—C12 are disordered over two positions (0.534 (6)/0.466 (6)). The hydrogen atom involved in the intermolecular hydrogen bond, H2, was freely refined.

### Crystal data

**Table:**

[Cd(C_12_H_27_O_3_SSi)_2_(C_3_H_4_N_2_)]	*Z* = 2
*M**_r_* = 739.45	*F*(000) = 780
Triclinic, *P*1	*D*_x_ = 1.29 Mg m^−^^3^
Hall symbol: -P 1	Mo *K*α radiation, λ = 0.71073 Å
*a* = 9.4445 (4) Å	Cell parameters from 7717 reflections
*b* = 12.7322 (4) Å	θ = 2.4–28.7°
*c* = 16.9153 (5) Å	µ = 0.78 mm^−^^1^
α = 109.098 (3)°	*T* = 120 K
β = 92.905 (3)°	Prism, colourless
γ = 96.050 (3)°	0.2 × 0.18 × 0.08 mm
*V* = 1903.53 (12) Å^3^	

### Data collection

**Table:**

Agilent Xcalibur Sapphire2 diffractometer	5935 reflections with *I* > 2σ(*I*)
Graphite monochromator	*R*_int_ = 0.025
Detector resolution: 8.1883 pixels mm^-1^	θ_max_ = 25.5°, θ_min_ = 2.4°
ω scans	*h* = −11→9
11378 measured reflections	*k* = −15→13
7083 independent reflections	*l* = −16→20

### Refinement

**Table:**

Refinement on *F*^2^	Primary atom site location: structure-invariant direct methods
Least-squares matrix: full	Secondary atom site location: difference Fourier map
*R*[*F*^2^ > 2σ(*F*^2^)] = 0.039	Hydrogen site location: inferred from neighbouring sites
*wR*(*F*^2^) = 0.103	H atoms treated by a mixture of independent and constrained refinement
*S* = 1.06	*w* = 1/[σ^2^(*F*_o_^2^) + (0.0596*P*)^2^ + 0.7954*P*] where *P* = (*F*_o_^2^ + 2*F*_c_^2^)/3
7083 reflections	(Δ/σ)_max_ = 0.002
414 parameters	Δρ_max_ = 1.11 e Å^−^^3^
0 restraints	Δρ_min_ = −0.65 e Å^−^^3^

### Special details

**Table:**

Geometry. All s.u.'s (except the s.u. in the dihedral angle between two l.s. planes) are estimated using the full covariance matrix. The cell s.u.'s are taken into account individually in the estimation of s.u.'s in distances, angles and torsion angles; correlations between s.u.'s in cell parameters are only used when they are defined by crystal symmetry. An approximate (isotropic) treatment of cell s.u.'s is used for estimating s.u.'s involving l.s. planes.
Refinement. Refinement of *F*^2^ against ALL reflections. The weighted *R*-factor *wR* and goodness of fit *S* are based on *F*^2^, conventional *R*-factors *R* are based on *F*, with *F* set to zero for negative *F*^2^. The threshold expression of *F*^2^ > 2σ(*F*^2^) is used only for calculating *R*-factors(gt) *etc*. and is not relevant to the choice of reflections for refinement. *R*-factors based on *F*^2^ are statistically about twice as large as those based on *F*, and *R*- factors based on ALL data will be even larger.

### Fractional atomic coordinates and isotropic or equivalent isotropic displacement parameters (Å^2^)

**Table:**

	*x*	*y*	*z*	*U*_iso_*/*U*_eq_	Occ. (<1)
Cd1	0.05597 (2)	0.985121 (18)	0.753064 (14)	0.02601 (9)	
S1	−0.17784 (8)	0.90765 (6)	0.77929 (5)	0.02601 (18)	
S2	0.17728 (9)	1.12410 (7)	0.69927 (5)	0.03058 (19)	
Si1	−0.18559 (8)	0.78496 (7)	0.66080 (5)	0.02040 (18)	
Si2	0.24411 (9)	1.22389 (7)	0.82244 (5)	0.02248 (19)	
O1	−0.0244 (2)	0.80690 (17)	0.63028 (13)	0.0253 (5)	
O2	−0.3100 (2)	0.78779 (17)	0.59191 (13)	0.0259 (5)	
O3	−0.2048 (2)	0.65667 (17)	0.66108 (13)	0.0267 (5)	
O4	0.1418 (2)	1.17162 (17)	0.88070 (12)	0.0240 (4)	
O5	0.4076 (2)	1.21018 (17)	0.85339 (13)	0.0242 (5)	
O6	0.2425 (2)	1.35733 (17)	0.84233 (14)	0.0290 (5)	
N1	0.2364 (3)	0.9134 (2)	0.80682 (16)	0.0269 (6)	
C1	0.0478 (4)	0.7388 (3)	0.5607 (2)	0.0313 (7)	
C2	0.1289 (4)	0.8205 (3)	0.5257 (2)	0.0390 (8)	
H2A	0.0609	0.8563	0.5007	0.058*	
H2B	0.1879	0.7801	0.4828	0.058*	
H2C	0.1904	0.8779	0.5712	0.058*	
C3	−0.0607 (4)	0.6570 (3)	0.4925 (2)	0.0508 (10)	
H3A	−0.1168	0.6074	0.5164	0.076*	
H3B	−0.0102	0.6121	0.4467	0.076*	
H3C	−0.1246	0.6991	0.4708	0.076*	
C4	0.1481 (5)	0.6780 (3)	0.5976 (3)	0.0474 (10)	
H4A	0.2158	0.7328	0.6411	0.071*	
H4B	0.2008	0.6326	0.5531	0.071*	
H4C	0.0926	0.6292	0.6223	0.071*	
C5	−0.3699 (3)	0.8759 (3)	0.5702 (2)	0.0301 (7)	
C6	−0.2526 (4)	0.9692 (3)	0.5720 (3)	0.0485 (10)	
H6A	−0.21	1.007	0.6298	0.073*	
H6B	−0.2938	1.0236	0.551	0.073*	
H6C	−0.1786	0.937	0.5363	0.073*	
C7	−0.4433 (4)	0.8186 (3)	0.4815 (2)	0.0470 (10)	
H7A	−0.3714	0.792	0.4424	0.07*	
H7B	−0.4939	0.8721	0.4644	0.07*	
H7C	−0.5118	0.7548	0.4808	0.07*	
C8	−0.4792 (4)	0.9197 (3)	0.6309 (2)	0.0377 (8)	
H8A	−0.5509	0.8575	0.6297	0.057*	
H8B	−0.5262	0.9755	0.6146	0.057*	
H8C	−0.4315	0.9544	0.6878	0.057*	
C9	−0.3262 (4)	0.5875 (3)	0.6738 (2)	0.0329 (8)	
C10	−0.4222 (8)	0.6548 (6)	0.7331 (5)	0.043 (2)	0.534 (6)
H10A	−0.371	0.6883	0.7892	0.064*	0.534 (6)
H10B	−0.5076	0.6057	0.7359	0.064*	0.534 (6)
H10C	−0.4505	0.7142	0.7131	0.064*	0.534 (6)
C11	−0.4105 (8)	0.5393 (5)	0.5828 (4)	0.0407 (19)	0.534 (6)
H11A	−0.436	0.6015	0.5653	0.061*	0.534 (6)
H11B	−0.4977	0.4911	0.5844	0.061*	0.534 (6)
H11C	−0.3497	0.4954	0.5427	0.061*	0.534 (6)
C12	−0.2692 (10)	0.4985 (7)	0.6966 (7)	0.068 (3)	0.534 (6)
H12A	−0.2071	0.4613	0.6543	0.101*	0.534 (6)
H12B	−0.3482	0.4437	0.6994	0.101*	0.534 (6)
H12C	−0.2139	0.5304	0.7515	0.101*	0.534 (6)
C10A	−0.4682 (8)	0.6233 (7)	0.6616 (5)	0.042 (2)	0.466 (6)
H10D	−0.4641	0.704	0.6911	0.064*	0.466 (6)
H10E	−0.5403	0.5825	0.6842	0.064*	0.466 (6)
H10F	−0.4938	0.6069	0.6015	0.064*	0.466 (6)
C11A	−0.3166 (11)	0.4659 (6)	0.6230 (5)	0.047 (2)	0.466 (6)
H11D	−0.333	0.4547	0.563	0.07*	0.466 (6)
H11E	−0.3891	0.4171	0.6386	0.07*	0.466 (6)
H11F	−0.2214	0.4475	0.6348	0.07*	0.466 (6)
C12A	−0.3010 (10)	0.5984 (6)	0.7706 (4)	0.041 (2)	0.466 (6)
H12D	−0.2174	0.5623	0.7787	0.062*	0.466 (6)
H12E	−0.3856	0.5617	0.7869	0.062*	0.466 (6)
H12F	−0.2847	0.6778	0.8053	0.062*	0.466 (6)
C13	0.1443 (4)	1.1983 (3)	0.97162 (19)	0.0288 (7)	
C14	0.2531 (4)	1.1374 (3)	1.0014 (2)	0.0401 (9)	
H14A	0.2298	1.0565	0.9723	0.06*	
H14B	0.2515	1.1535	1.0621	0.06*	
H14C	0.3486	1.1627	0.9892	0.06*	
C15	0.1815 (6)	1.3247 (3)	1.0166 (2)	0.0560 (12)	
H15A	0.179	1.3414	1.0773	0.084*	
H15B	0.1119	1.3643	0.9966	0.084*	
H15C	0.2775	1.3492	1.0047	0.084*	
C16	−0.0053 (4)	1.1580 (4)	0.9872 (2)	0.0496 (10)	
H16A	−0.0314	1.0791	0.9525	0.074*	
H16B	−0.0733	1.2032	0.9725	0.074*	
H16C	−0.0078	1.1656	1.0467	0.074*	
C17	0.5467 (3)	1.2632 (3)	0.8437 (2)	0.0283 (7)	
C18	0.6535 (4)	1.1934 (3)	0.8649 (3)	0.0432 (9)	
H18A	0.6378	1.1176	0.8236	0.065*	
H18B	0.641	1.1896	0.9211	0.065*	
H18C	0.7508	1.2279	0.8637	0.065*	
C19	0.5714 (4)	1.3828 (3)	0.9049 (3)	0.0458 (9)	
H19A	0.6643	1.4194	0.8979	0.069*	
H19B	0.5704	1.3817	0.9625	0.069*	
H19C	0.4956	1.4242	0.8936	0.069*	
C20	0.5536 (4)	1.2608 (3)	0.7534 (2)	0.0412 (9)	
H20A	0.5294	1.1836	0.7151	0.062*	
H20B	0.6505	1.2899	0.7459	0.062*	
H20C	0.4854	1.3076	0.7411	0.062*	
C21	0.1447 (4)	1.4231 (3)	0.8169 (3)	0.0405 (9)	
C22	0.1711 (5)	1.5389 (3)	0.8845 (3)	0.0542 (11)	
H22A	0.1517	1.532	0.9391	0.081*	
H22B	0.1077	1.5879	0.8709	0.081*	
H22C	0.2709	1.5709	0.8869	0.081*	
C23	0.1803 (6)	1.4289 (4)	0.7328 (3)	0.0647 (13)	
H23A	0.2823	1.4555	0.7355	0.097*	
H23B	0.1235	1.4809	0.7178	0.097*	
H23C	0.1584	1.3542	0.6903	0.097*	
C24	−0.0101 (4)	1.3714 (4)	0.8152 (4)	0.0636 (14)	
H24A	−0.0282	1.2984	0.7704	0.095*	
H24B	−0.0756	1.4211	0.8047	0.095*	
H24C	−0.0253	1.3615	0.8693	0.095*	
N2	0.3708 (3)	0.9677 (2)	0.82922 (17)	0.0279 (6)	
C25	0.2363 (4)	0.8215 (3)	0.8284 (2)	0.0345 (8)	
H25	0.1562	0.7658	0.8195	0.041*	
C27	0.4531 (4)	0.9137 (3)	0.8654 (2)	0.0371 (8)	
H27	0.5506	0.9367	0.8866	0.045*	
C26	0.3699 (4)	0.8187 (3)	0.8660 (2)	0.0433 (9)	
H26	0.3976	0.763	0.8875	0.052*	
H2	0.386 (5)	1.033 (3)	0.828 (2)	0.049 (12)*	

### Atomic displacement parameters (Å^2^)

**Table:**

	*U*^11^	*U*^22^	*U*^33^	*U*^12^	*U*^13^	*U*^23^
Cd1	0.02004 (13)	0.02765 (14)	0.03054 (14)	−0.00224 (9)	−0.00191 (9)	0.01224 (10)
S1	0.0216 (4)	0.0287 (4)	0.0268 (4)	0.0005 (3)	0.0043 (3)	0.0086 (3)
S2	0.0315 (4)	0.0341 (4)	0.0266 (4)	−0.0069 (4)	−0.0033 (3)	0.0150 (3)
Si1	0.0154 (4)	0.0220 (4)	0.0259 (4)	0.0007 (3)	0.0014 (3)	0.0115 (3)
Si2	0.0189 (4)	0.0235 (4)	0.0275 (4)	−0.0002 (3)	−0.0005 (3)	0.0131 (4)
O1	0.0174 (11)	0.0269 (11)	0.0283 (11)	0.0002 (9)	0.0038 (9)	0.0056 (9)
O2	0.0240 (11)	0.0239 (11)	0.0323 (11)	0.0006 (9)	−0.0039 (9)	0.0146 (9)
O3	0.0234 (11)	0.0243 (11)	0.0370 (12)	0.0017 (9)	0.0011 (9)	0.0169 (10)
O4	0.0195 (11)	0.0291 (11)	0.0258 (11)	0.0003 (9)	0.0018 (9)	0.0135 (9)
O5	0.0178 (11)	0.0269 (11)	0.0314 (11)	0.0009 (9)	0.0009 (9)	0.0153 (9)
O6	0.0236 (12)	0.0274 (11)	0.0406 (13)	0.0015 (9)	0.0004 (10)	0.0183 (10)
N1	0.0236 (14)	0.0262 (13)	0.0309 (14)	0.0003 (11)	0.0002 (11)	0.0107 (11)
C1	0.0248 (17)	0.0340 (17)	0.0304 (17)	0.0031 (14)	0.0074 (14)	0.0042 (14)
C2	0.035 (2)	0.047 (2)	0.0370 (19)	0.0085 (17)	0.0141 (16)	0.0142 (17)
C3	0.038 (2)	0.054 (2)	0.041 (2)	−0.0061 (19)	0.0101 (17)	−0.0090 (18)
C4	0.053 (3)	0.039 (2)	0.052 (2)	0.0215 (19)	0.0159 (19)	0.0106 (18)
C5	0.0244 (17)	0.0327 (17)	0.0406 (19)	0.0054 (14)	−0.0014 (14)	0.0225 (15)
C6	0.032 (2)	0.048 (2)	0.086 (3)	0.0041 (17)	0.001 (2)	0.051 (2)
C7	0.047 (2)	0.060 (2)	0.041 (2)	0.018 (2)	−0.0084 (18)	0.0253 (19)
C8	0.0254 (18)	0.0387 (19)	0.051 (2)	0.0081 (15)	−0.0032 (16)	0.0174 (17)
C9	0.0361 (19)	0.0244 (16)	0.0384 (19)	−0.0038 (14)	0.0042 (15)	0.0131 (15)
C10	0.040 (4)	0.042 (4)	0.045 (5)	−0.013 (3)	0.011 (3)	0.018 (3)
C11	0.034 (4)	0.031 (3)	0.049 (4)	−0.014 (3)	−0.001 (3)	0.008 (3)
C12	0.071 (6)	0.047 (5)	0.109 (9)	0.010 (4)	0.012 (6)	0.058 (6)
C10A	0.034 (4)	0.048 (5)	0.050 (5)	−0.012 (4)	0.009 (4)	0.027 (4)
C11A	0.084 (7)	0.025 (4)	0.025 (4)	−0.005 (4)	0.011 (4)	0.002 (3)
C12A	0.078 (6)	0.022 (4)	0.022 (4)	−0.002 (4)	−0.002 (4)	0.009 (3)
C13	0.0302 (18)	0.0324 (17)	0.0252 (16)	0.0038 (14)	0.0044 (13)	0.0114 (14)
C14	0.041 (2)	0.059 (2)	0.0245 (17)	0.0125 (18)	0.0010 (15)	0.0170 (17)
C15	0.090 (4)	0.036 (2)	0.038 (2)	0.009 (2)	0.017 (2)	0.0053 (18)
C16	0.033 (2)	0.082 (3)	0.039 (2)	0.000 (2)	0.0078 (17)	0.030 (2)
C17	0.0173 (15)	0.0312 (17)	0.0366 (18)	−0.0024 (13)	−0.0003 (13)	0.0139 (14)
C18	0.0218 (18)	0.044 (2)	0.069 (3)	0.0003 (16)	−0.0036 (17)	0.029 (2)
C19	0.030 (2)	0.038 (2)	0.058 (2)	−0.0063 (16)	−0.0026 (18)	0.0054 (18)
C20	0.0273 (19)	0.057 (2)	0.043 (2)	−0.0030 (17)	0.0040 (16)	0.0228 (18)
C21	0.034 (2)	0.0340 (18)	0.062 (2)	0.0080 (16)	−0.0054 (17)	0.0276 (18)
C22	0.053 (3)	0.0282 (19)	0.086 (3)	0.0116 (18)	0.002 (2)	0.024 (2)
C23	0.077 (3)	0.063 (3)	0.070 (3)	0.009 (3)	−0.009 (3)	0.047 (3)
C24	0.027 (2)	0.049 (2)	0.124 (4)	0.0074 (19)	−0.006 (2)	0.042 (3)
N2	0.0213 (14)	0.0289 (14)	0.0368 (15)	0.0008 (12)	−0.0002 (11)	0.0165 (13)
C25	0.0345 (19)	0.0308 (17)	0.043 (2)	−0.0001 (15)	0.0046 (16)	0.0198 (16)
C27	0.0251 (18)	0.045 (2)	0.048 (2)	0.0105 (16)	−0.0010 (15)	0.0238 (17)
C26	0.043 (2)	0.040 (2)	0.061 (2)	0.0089 (17)	0.0015 (18)	0.0344 (19)

### Geometric parameters (Å, º)

**Table:**

Cd1—N1	2.298 (3)	C11—H11C	0.98
Cd1—O1	2.536 (2)	C12—H12A	0.98
Cd1—O4	2.649 (2)	C12—H12B	0.98
Cd1—S1	2.4438 (8)	C12—H12C	0.98
Cd1—S2	2.4503 (8)	C10A—H10D	0.98
Cd1—Si1	3.1538 (8)	C10A—H10E	0.98
Si1—S1	2.0917 (11)	C10A—H10F	0.98
Si2—S2	2.0781 (12)	C11A—H11D	0.98
Si1—O2	1.624 (2)	C11A—H11E	0.98
Si1—O3	1.626 (2)	C11A—H11F	0.98
Si1—O1	1.658 (2)	C12A—H12D	0.98
Si2—O6	1.622 (2)	C12A—H12E	0.98
Si2—O5	1.650 (2)	C12A—H12F	0.98
Si2—O4	1.655 (2)	C13—C14	1.511 (5)
O1—C1	1.463 (4)	C13—C16	1.517 (5)
O2—C5	1.448 (4)	C13—C15	1.530 (5)
O3—C9	1.439 (4)	C14—H14A	0.98
O4—C13	1.461 (4)	C14—H14B	0.98
O5—C17	1.455 (4)	C14—H14C	0.98
O6—C21	1.448 (4)	C15—H15A	0.98
N1—C25	1.335 (4)	C15—H15B	0.98
N1—N2	1.352 (4)	C15—H15C	0.98
C1—C2	1.516 (5)	C16—H16A	0.98
C1—C4	1.516 (5)	C16—H16B	0.98
C1—C3	1.528 (5)	C16—H16C	0.98
C2—H2A	0.98	C17—C18	1.516 (5)
C2—H2B	0.98	C17—C19	1.521 (5)
C2—H2C	0.98	C17—C20	1.522 (5)
C3—H3A	0.98	C18—H18A	0.98
C3—H3B	0.98	C18—H18B	0.98
C3—H3C	0.98	C18—H18C	0.98
C4—H4A	0.98	C19—H19A	0.98
C4—H4B	0.98	C19—H19B	0.98
C4—H4C	0.98	C19—H19C	0.98
C5—C8	1.513 (5)	C20—H20A	0.98
C5—C7	1.528 (5)	C20—H20B	0.98
C5—C6	1.528 (5)	C20—H20C	0.98
C6—H6A	0.98	C21—C23	1.502 (6)
C6—H6B	0.98	C21—C22	1.528 (5)
C6—H6C	0.98	C21—C24	1.533 (5)
C7—H7A	0.98	C22—H22A	0.98
C7—H7B	0.98	C22—H22B	0.98
C7—H7C	0.98	C22—H22C	0.98
C8—H8A	0.98	C23—H23A	0.98
C8—H8B	0.98	C23—H23B	0.98
C8—H8C	0.98	C23—H23C	0.98
C9—C12	1.456 (8)	C24—H24A	0.98
C9—C10A	1.486 (9)	C24—H24B	0.98
C9—C10	1.502 (8)	C24—H24C	0.98
C9—C11A	1.520 (8)	N2—C27	1.338 (4)
C9—C11	1.591 (7)	N2—H2	0.83 (4)
C9—C12A	1.601 (7)	C25—C26	1.393 (5)
C10—H10A	0.98	C25—H25	0.95
C10—H10B	0.98	C27—C26	1.375 (5)
C10—H10C	0.98	C27—H27	0.95
C11—H11A	0.98	C26—H26	0.95
C11—H11B	0.98		
			
N1—Cd1—O1	94.98 (8)	C11A—C9—C12A	106.4 (5)
N1—Cd1—O4	84.89 (8)	C11—C9—C12A	153.7 (4)
N1—Cd1—S1	110.92 (7)	C9—C10—H10A	109.5
N1—Cd1—S2	105.14 (7)	C9—C10—H10B	109.5
O1—Cd1—O4	179.52 (6)	C9—C10—H10C	109.5
S1—Cd1—O1	72.84 (5)	C9—C11—H11A	109.5
S1—Cd1—O4	107.64 (5)	C9—C11—H11B	109.5
S2—Cd1—O1	108.45 (5)	C9—C11—H11C	109.5
S2—Cd1—O4	71.15 (5)	C9—C12—H12A	109.5
S1—Cd1—S2	143.70 (3)	C9—C12—H12B	109.5
N1—Cd1—Si1	107.76 (7)	C9—C12—H12C	109.5
S1—Cd1—Si1	41.51 (2)	C9—C10A—H10D	109.5
S2—Cd1—Si1	128.78 (2)	C9—C10A—H10E	109.5
O1—Cd1—Si1	31.56 (5)	H10D—C10A—H10E	109.5
O4—Cd1—Si1	148.91 (5)	C9—C10A—H10F	109.5
Si1—S1—Cd1	87.75 (3)	H10D—C10A—H10F	109.5
Si2—S2—Cd1	88.70 (4)	H10E—C10A—H10F	109.5
O2—Si1—O3	105.07 (11)	C9—C11A—H11D	109.5
O2—Si1—O1	111.27 (11)	C9—C11A—H11E	109.5
O3—Si1—O1	106.14 (11)	H11D—C11A—H11E	109.5
O2—Si1—S1	115.77 (9)	C9—C11A—H11F	109.5
O3—Si1—S1	114.79 (9)	H11D—C11A—H11F	109.5
O1—Si1—S1	103.54 (8)	H11E—C11A—H11F	109.5
O2—Si1—Cd1	124.75 (8)	C9—C12A—H12D	109.5
O3—Si1—Cd1	129.82 (8)	C9—C12A—H12E	109.5
O1—Si1—Cd1	53.20 (7)	H12D—C12A—H12E	109.5
S1—Si1—Cd1	50.74 (3)	C9—C12A—H12F	109.5
O6—Si2—O5	105.57 (11)	H12D—C12A—H12F	109.5
O6—Si2—O4	113.49 (12)	H12E—C12A—H12F	109.5
O5—Si2—O4	103.78 (11)	O4—C13—C14	110.0 (3)
O6—Si2—S2	115.13 (9)	O4—C13—C16	105.4 (3)
O5—Si2—S2	113.55 (9)	C14—C13—C16	110.6 (3)
O4—Si2—S2	104.93 (8)	O4—C13—C15	110.6 (3)
C1—O1—Si1	131.05 (19)	C14—C13—C15	109.4 (3)
C1—O1—Cd1	133.45 (17)	C16—C13—C15	110.8 (3)
Si1—O1—Cd1	95.24 (9)	C13—C14—H14A	109.5
C5—O2—Si1	134.6 (2)	C13—C14—H14B	109.5
C9—O3—Si1	131.5 (2)	H14A—C14—H14B	109.5
C13—O4—Si2	131.51 (19)	C13—C14—H14C	109.5
C13—O4—Cd1	132.44 (17)	H14A—C14—H14C	109.5
Si2—O4—Cd1	92.19 (9)	H14B—C14—H14C	109.5
C17—O5—Si2	131.52 (18)	C13—C15—H15A	109.5
C21—O6—Si2	133.6 (2)	C13—C15—H15B	109.5
C25—N1—N2	105.3 (3)	H15A—C15—H15B	109.5
C25—N1—Cd1	131.5 (2)	C13—C15—H15C	109.5
N2—N1—Cd1	122.98 (19)	H15A—C15—H15C	109.5
O1—C1—C2	106.0 (3)	H15B—C15—H15C	109.5
O1—C1—C4	106.7 (3)	C13—C16—H16A	109.5
C2—C1—C4	111.6 (3)	C13—C16—H16B	109.5
O1—C1—C3	110.8 (3)	H16A—C16—H16B	109.5
C2—C1—C3	109.8 (3)	C13—C16—H16C	109.5
C4—C1—C3	111.7 (3)	H16A—C16—H16C	109.5
C1—C2—H2A	109.5	H16B—C16—H16C	109.5
C1—C2—H2B	109.5	O5—C17—C18	105.1 (2)
H2A—C2—H2B	109.5	O5—C17—C19	109.1 (3)
C1—C2—H2C	109.5	C18—C17—C19	110.9 (3)
H2A—C2—H2C	109.5	O5—C17—C20	109.9 (3)
H2B—C2—H2C	109.5	C18—C17—C20	110.6 (3)
C1—C3—H3A	109.5	C19—C17—C20	111.2 (3)
C1—C3—H3B	109.5	C17—C18—H18A	109.5
H3A—C3—H3B	109.5	C17—C18—H18B	109.5
C1—C3—H3C	109.5	H18A—C18—H18B	109.5
H3A—C3—H3C	109.5	C17—C18—H18C	109.5
H3B—C3—H3C	109.5	H18A—C18—H18C	109.5
C1—C4—H4A	109.5	H18B—C18—H18C	109.5
C1—C4—H4B	109.5	C17—C19—H19A	109.5
H4A—C4—H4B	109.5	C17—C19—H19B	109.5
C1—C4—H4C	109.5	H19A—C19—H19B	109.5
H4A—C4—H4C	109.5	C17—C19—H19C	109.5
H4B—C4—H4C	109.5	H19A—C19—H19C	109.5
O2—C5—C8	108.3 (3)	H19B—C19—H19C	109.5
O2—C5—C7	104.9 (3)	C17—C20—H20A	109.5
C8—C5—C7	110.1 (3)	C17—C20—H20B	109.5
O2—C5—C6	110.9 (3)	H20A—C20—H20B	109.5
C8—C5—C6	111.5 (3)	C17—C20—H20C	109.5
C7—C5—C6	110.9 (3)	H20A—C20—H20C	109.5
C5—C6—H6A	109.5	H20B—C20—H20C	109.5
C5—C6—H6B	109.5	O6—C21—C23	108.3 (3)
H6A—C6—H6B	109.5	O6—C21—C22	105.4 (3)
C5—C6—H6C	109.5	C23—C21—C22	111.2 (3)
H6A—C6—H6C	109.5	O6—C21—C24	110.0 (3)
H6B—C6—H6C	109.5	C23—C21—C24	112.3 (4)
C5—C7—H7A	109.5	C22—C21—C24	109.4 (4)
C5—C7—H7B	109.5	C21—C22—H22A	109.5
H7A—C7—H7B	109.5	C21—C22—H22B	109.5
C5—C7—H7C	109.5	H22A—C22—H22B	109.5
H7A—C7—H7C	109.5	C21—C22—H22C	109.5
H7B—C7—H7C	109.5	H22A—C22—H22C	109.5
C5—C8—H8A	109.5	H22B—C22—H22C	109.5
C5—C8—H8B	109.5	C21—C23—H23A	109.5
H8A—C8—H8B	109.5	C21—C23—H23B	109.5
C5—C8—H8C	109.5	H23A—C23—H23B	109.5
H8A—C8—H8C	109.5	C21—C23—H23C	109.5
H8B—C8—H8C	109.5	H23A—C23—H23C	109.5
O3—C9—C12	106.2 (5)	H23B—C23—H23C	109.5
O3—C9—C10A	115.9 (4)	C21—C24—H24A	109.5
C12—C9—C10A	137.9 (5)	C21—C24—H24B	109.5
O3—C9—C10	112.2 (3)	H24A—C24—H24B	109.5
C12—C9—C10	116.4 (6)	C21—C24—H24C	109.5
C10A—C9—C10	46.8 (4)	H24A—C24—H24C	109.5
O3—C9—C11A	108.8 (4)	H24B—C24—H24C	109.5
C12—C9—C11A	48.3 (5)	C27—N2—N1	111.8 (3)
C10A—C9—C11A	113.7 (6)	C27—N2—H2	129 (3)
C10—C9—C11A	138.9 (5)	N1—N2—H2	118 (3)
O3—C9—C11	102.5 (3)	N1—C25—C26	110.5 (3)
C12—C9—C11	110.8 (6)	N1—C25—H25	124.7
C10A—C9—C11	61.5 (5)	C26—C25—H25	124.7
C10—C9—C11	107.9 (5)	N2—C27—C26	107.0 (3)
C11A—C9—C11	63.1 (5)	N2—C27—H27	126.5
O3—C9—C12A	103.8 (4)	C26—C27—H27	126.5
C12—C9—C12A	60.0 (5)	C27—C26—C25	105.4 (3)
C10A—C9—C12A	107.2 (5)	C27—C26—H26	127.3
C10—C9—C12A	62.8 (5)	C25—C26—H26	127.3
			
N1—Cd1—S1—Si1	−93.48 (8)	S2—Si2—O4—Cd1	14.63 (8)
S2—Cd1—S1—Si1	93.33 (5)	N1—Cd1—O4—C13	−63.7 (2)
O1—Cd1—S1—Si1	−4.67 (6)	S1—Cd1—O4—C13	46.6 (2)
O4—Cd1—S1—Si1	175.29 (5)	S2—Cd1—O4—C13	−171.7 (2)
N1—Cd1—S2—Si2	−69.02 (8)	Si1—Cd1—O4—C13	52.7 (3)
S1—Cd1—S2—Si2	104.39 (5)	N1—Cd1—O4—Si2	95.28 (10)
O1—Cd1—S2—Si2	−169.69 (6)	S1—Cd1—O4—Si2	−154.37 (7)
O4—Cd1—S2—Si2	10.03 (5)	S2—Cd1—O4—Si2	−12.64 (7)
Si1—Cd1—S2—Si2	162.46 (3)	Si1—Cd1—O4—Si2	−148.32 (6)
Cd1—S1—Si1—O2	−115.01 (9)	O6—Si2—O5—C17	−42.6 (3)
Cd1—S1—Si1—O3	122.25 (9)	O4—Si2—O5—C17	−162.3 (2)
Cd1—S1—Si1—O1	7.03 (8)	S2—Si2—O5—C17	84.4 (3)
N1—Cd1—Si1—O2	−161.55 (12)	O5—Si2—O6—C21	166.1 (3)
S1—Cd1—Si1—O2	96.68 (11)	O4—Si2—O6—C21	−80.9 (3)
S2—Cd1—Si1—O2	−34.02 (11)	S2—Si2—O6—C21	40.1 (3)
O1—Cd1—Si1—O2	−91.86 (14)	S1—Cd1—N1—C25	19.6 (3)
O4—Cd1—Si1—O2	87.97 (14)	S2—Cd1—N1—C25	−164.6 (3)
N1—Cd1—Si1—O3	10.42 (13)	O1—Cd1—N1—C25	−53.9 (3)
S1—Cd1—Si1—O3	−91.35 (12)	O4—Cd1—N1—C25	126.6 (3)
S2—Cd1—Si1—O3	137.95 (11)	Si1—Cd1—N1—C25	−24.4 (3)
O1—Cd1—Si1—O3	80.11 (14)	S1—Cd1—N1—N2	−155.0 (2)
O4—Cd1—Si1—O3	−100.06 (14)	S2—Cd1—N1—N2	20.8 (2)
N1—Cd1—Si1—O1	−69.69 (11)	O1—Cd1—N1—N2	131.5 (2)
S1—Cd1—Si1—O1	−171.46 (10)	O4—Cd1—N1—N2	−48.0 (2)
S2—Cd1—Si1—O1	57.84 (10)	Si1—Cd1—N1—N2	161.0 (2)
O4—Cd1—Si1—O1	179.83 (12)	Si1—O1—C1—C2	140.9 (2)
N1—Cd1—Si1—S1	101.77 (8)	Cd1—O1—C1—C2	−46.4 (4)
S2—Cd1—Si1—S1	−130.70 (4)	Si1—O1—C1—C4	−100.0 (3)
O1—Cd1—Si1—S1	171.46 (10)	Cd1—O1—C1—C4	72.8 (3)
O4—Cd1—Si1—S1	−8.71 (10)	Si1—O1—C1—C3	21.8 (4)
Cd1—S2—Si2—O6	−141.34 (10)	Cd1—O1—C1—C3	−165.5 (2)
Cd1—S2—Si2—O5	96.80 (9)	Si1—O2—C5—C8	−82.0 (3)
Cd1—S2—Si2—O4	−15.84 (9)	Si1—O2—C5—C7	160.5 (3)
O2—Si1—O1—C1	−67.1 (3)	Si1—O2—C5—C6	40.7 (4)
O3—Si1—O1—C1	46.7 (3)	Si1—O3—C9—C12	−160.2 (5)
S1—Si1—O1—C1	167.9 (2)	Si1—O3—C9—C10A	19.4 (6)
Cd1—Si1—O1—C1	174.7 (3)	Si1—O3—C9—C10	−32.0 (5)
O2—Si1—O1—Cd1	118.20 (10)	Si1—O3—C9—C11A	149.0 (4)
O3—Si1—O1—Cd1	−128.03 (10)	Si1—O3—C9—C11	83.5 (4)
S1—Si1—O1—Cd1	−6.79 (8)	Si1—O3—C9—C12A	−97.9 (4)
N1—Cd1—O1—C1	−58.2 (3)	Si2—O4—C13—C14	−82.8 (3)
S1—Cd1—O1—C1	−168.6 (3)	Cd1—O4—C13—C14	68.6 (3)
S2—Cd1—O1—C1	49.6 (2)	Si2—O4—C13—C16	158.0 (2)
Si1—Cd1—O1—C1	−174.5 (3)	Cd1—O4—C13—C16	−50.6 (3)
N1—Cd1—O1—Si1	116.29 (10)	Si2—O4—C13—C15	38.2 (4)
S1—Cd1—O1—Si1	5.91 (7)	Cd1—O4—C13—C15	−170.4 (2)
S2—Cd1—O1—Si1	−135.92 (7)	Si2—O5—C17—C18	−165.4 (2)
O3—Si1—O2—C5	167.4 (3)	Si2—O5—C17—C19	75.7 (3)
O1—Si1—O2—C5	−78.2 (3)	Si2—O5—C17—C20	−46.4 (4)
S1—Si1—O2—C5	39.7 (3)	Si2—O6—C21—C23	−84.2 (4)
Cd1—Si1—O2—C5	−19.0 (3)	Si2—O6—C21—C22	156.7 (3)
O2—Si1—O3—C9	−55.0 (3)	Si2—O6—C21—C24	38.8 (5)
O1—Si1—O3—C9	−172.9 (3)	C25—N1—N2—C27	−1.2 (4)
S1—Si1—O3—C9	73.4 (3)	Cd1—N1—N2—C27	174.6 (2)
Cd1—Si1—O3—C9	131.9 (2)	N2—N1—C25—C26	1.1 (4)
O6—Si2—O4—C13	−59.5 (3)	Cd1—N1—C25—C26	−174.2 (2)
O5—Si2—O4—C13	54.6 (3)	N1—N2—C27—C26	0.8 (4)
S2—Si2—O4—C13	174.0 (2)	N2—C27—C26—C25	−0.1 (4)
O6—Si2—O4—Cd1	141.15 (10)	N1—C25—C26—C27	−0.7 (4)
O5—Si2—O4—Cd1	−104.78 (9)		

### Hydrogen-bond geometry (Å, º)

**Table:**

*D*—H···*A*	*D*—H	H···*A*	*D*···*A*	*D*—H···*A*
N2—H2···O5	0.83 (4)	2.14 (4)	2.959 (3)	167 (4)
